# The Adoption of Social Media to Recruit Participants for the Cool Runnings Randomized Controlled Trial in Australia

**DOI:** 10.2196/resprot.8189

**Published:** 2017-10-24

**Authors:** Jacqueline D Burgess, Roy M Kimble, Kerrianne Watt, Cate M Cameron

**Affiliations:** ^1^ Centre for Children's Burns and Trauma Research University of Queensland South Brisbane Australia; ^2^ Wound Management Innovation Cooperative Research Centre Brisbane Australia; ^3^ Pegg Leditschke Children’s Burns Centre Lady Cilento Childen’s Hospital Brisbane Australia; ^4^ Urology Burns & Trauma Unit, Department of Paediatric Surgery Lady Cilento Children's Hospital Brisbane Australia; ^5^ College of Public Health, Medical and Veterinary Sciences James Cook University Townsville Australia; ^6^ The Hopkins Centre, Menzies Health Institute Queensland Griffith University Meadowbrook Australia

**Keywords:** social media, online recruitment, burn prevention, methods, randomized controlled trial

## Abstract

**Background:**

Using social media to recruit specific populations for research studies is gaining popularity. Given that mothers of young children are the most active on social media, and young children are the most at risk of preventable burn injuries, social media was used to recruit mothers of young children to a burn prevention intervention.

**Objective:**

The aim of this paper was to describe the social media recruitment methods used to enroll mothers of young children to the app-based burn prevention intervention Cool Runnings.

**Methods:**

Participants were recruited via paid Facebook and Instagram advertisements to a 2-group, parallel, single-blinded, randomized controlled trial (RCT). The advertisements were targeted at women 18 years and older, living in Queensland, Australia, with at least 1 child aged 5 to 12 months at the time of recruitment.

**Results:**

Over the 30-day recruitment period from January to February 2016, Facebook and Instagram advertisements reached 65,268 people, generating 2573 link clicks, 1161 app downloads, and 498 enrolled participants to the Cool Runnings RCT. The cost per enrolled participant was Aus $13.08. Saturdays were the most effective day of the week for advertising results. The most popular time of day for enrolments was between 5 to 11 PM. This recruitment strategy campaign resulted in a broad reach of participants from regional, rural, and remote Queensland. Participants were representative of the population in regard to age and education levels.

**Conclusions:**

To our knowledge, this is the first use of social media recruitment for an injury prevention campaign. This recruitment method resulted in the rapid and cost-effective recruitment of participants with social, geographic, and economic diversity that were largely representative of the population.

## Introduction

More than half of the world’s population now has access to the Internet, and 2.8 billion people actively use social media [1]. Social media has become an integral part of modern society. It is more than just a place for friends to connect socially; it is used for politics, education, entertainment, shopping, and health. While commercial companies were quick to see the potential of social media to reach and interact with large targeted populations, researchers were slow adopters. Increasingly, social media platforms, such as Facebook, are being used to recruit participants for health and medical research [2,3].

Facebook’s ability to target advertising to specific demographics from its diversity of users offers researchers an opportunity to recruit populations that can be hard to access via traditional recruitment methods, including economically disadvantaged and geographically remote populations [3,4,5]. Globally, social media users have increased by 21% since January 2016 [1]. Facebook alone has 2 billion monthly users [6]. In Australia, approximately 70% of the population actively uses Facebook and the largest demographic are women aged 25 to 34 years [5]. Mothers with children under 5 years of age are the most active on social media [5,7].

Burns are the 5th most common cause of non-fatal childhood injuries globally [8]. Most burns to children under the age of 4 are scalds, predominantly from hot beverages [9-11]. In Australia, hot beverage scalds account for 1 in 5 burns to children—a figure that has remained the same for the past 15 years [9]. Added to this issue is the low use of correct burn first aid at the scene, despite strong evidence that burn first aid applied within 3 hours of the burn occurring provides pain relief and leads to less scarring, fewer surgical interventions, and shorter hospital stays [12]. Beyond the pain, itching, and scarring that can result from these injuries, there are also the long-term effects burns take on both the child and family. The frequent hospital visits/admissions for ongoing scar management, coping with changes in appearance, and people’s reaction to the scar can lead to social and psychological problems. There are also financial costs both to the family when parents take time off work to care for the injured child and their continuing rehabilitation needs and the cost to the healthcare system. In the United Kingdom, they have reported the cost of treating a minor scald as £1850 (US $2400) [13]. In children, this figure is higher as scar management and surgical procedures continue until they stop growing.

The high physical, emotional, and financial burdens associated with hot beverage scalds make it an important public health issue. Increasing awareness regarding burn severity and frequency of hot beverage scalds, as well as correct burn first aid, is an important step in reducing the burden of this injury [14-16]. To date, public health interventions and injury prevention are areas where technology has been underutilized.

In light of the popularity of social media in mothers of young children and evidence of social media’s broad reach, cost efficiencies, and capacity for targeting specific populations, social media was used to recruit mothers to the app-based burn prevention intervention Cool Runnings. The Cool Runnings app was used as the channel for delivering the 2-group, parallel, single-blinded, randomized controlled trial (RCT) over the 6-month intervention period (described in detail elsewhere). The purpose of this paper was to describe the use of social media as a tool for recruiting mothers of young children to this RCT.

## Methods

### Participants

Participants were recruited over a 30-day period for a 2-group, parallel, single-blinded RCT—Cool Runnings—aimed at changing knowledge about burn risks and correct burn first aid treatment in mothers of young children. The protocol for this study was published previously [17]. The inclusion criteria for this study were females aged 18 years and older with at least 1 child aged 5 to 12 months, who owned a mobile phone, and resided in the state of Queensland, Australia. Participants were recruited through Facebook and Instagram advertisements between January and February 2016. The state of Queensland is 1,852,642 km^2^ (approximately 2½ times the size of Texas, or 3 times the size of France) with a population of 4.9 million.

### Facebook Recruitment

**Figure 1:**
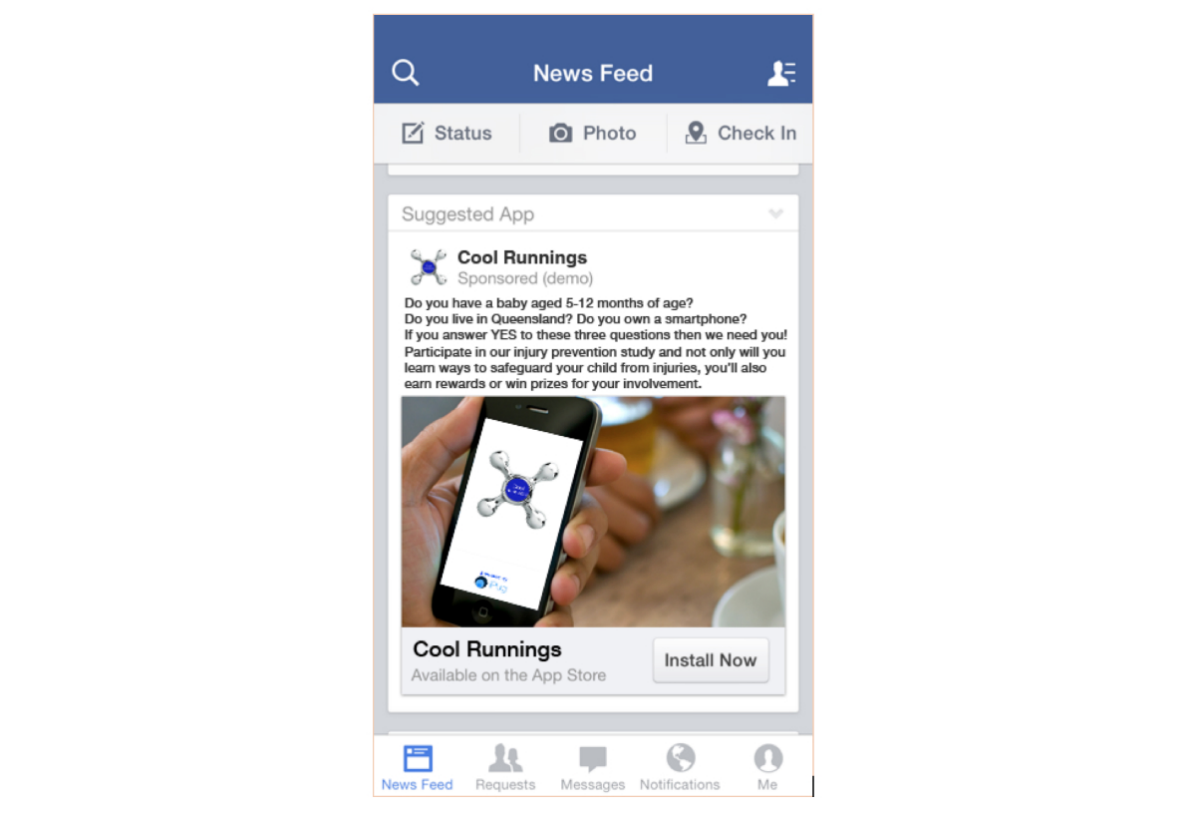
Example incentive-based recruitment advertisement used on Facebook.

Facebook and Instagram advertisements were directed to the target group described above. Facebook’s Audience Insights tool was used to better understand the social and psychological triggers of the target group. Demographic filtering showed the audiences “liked” pages, lifestyle factors, education, job titles, and frequency of activities, and this information informed the approach, messaging, and strategies for recruitment on the 2 platforms. Targeted, persuasive ad copy was developed for the Facebook and Instagram advertisements. Ad sets used 2 message themes: incentive-based and emotive-based. The incentive-based messages leveraged the ability to earn rewards and win prizes to drive recruitment. An example of one of the incentive-based messages is shown in Figure 1. The emotive-based messages created an emotional response in potential participants and called out the “greater good” of participating in a study aimed at keeping children safe. An example of an emotive-based message is:

21 children die each week in Australia from preventable injuries! Thousands more are hospitalized. With your help we can reduce this. Download the Cool Runnings app and learn ways to protect your child and other children from preventable injuries.

Altruistic and incentive-based messages are recognized as influential motivators in behavioral studies [18,19]. Messages that arouse emotions in potential participants and make an impact are more likely to get their attention and motivate them to take action [20]. These messages also aimed to raise mothers’ awareness of the threat of injuries in young children, combined with an efficacy component to learn how to prevent these injuries. Messages that combine threat and self-efficacy components are more effective than just threat and/or fear based messages [21].

A total of 45 advertisement sets were developed, each containing different combinations (ie, device type, visual elements, message theme, and ad placement). The variables tested were Apple (iOS) versus Android, video versus photo versus carouse, emotive versus incentive, and Facebook mobile newsfeed versus Instagram advertisement.

In total, 32 adaptations of the advertising copy were divided across the variables listed above. From the advertisements, interested individuals could click on an embedded link taking them directly to the Cool Runnings app in the Google Play or Apple App Store.

### Outcome Measures

Instagram is owned by Facebook, which allows the management of advertising campaigns and/or ad placement on both platforms from Facebook Ads Manager portal. This portal provides the following advertisement metrics: (1) impressions, number of times ads were shown; (2) reach, number of individual people who saw the ads; (3) link clicks, number of people who saw the ad and clicked through to download the app; (4) video views, number of times video viewed for 3 seconds or more; and (5) costs per 1000 impressions, per reach, per link click.

The number of Apple and Android app downloads resulting from the advertisement link clicks and the subsequent individuals who consented to participate in the study were calculated to determine the cost per participant.

### Study Enrollment

Once the app was downloaded, individuals were provided with additional information about the study and given the opportunity to consent to participate. Participants completed a 19-item questionnaire detailing demographic factors (such as education level, age of youngest child, number of children, marital status, and smoking status) and level of child burn risk knowledge and burn first aid knowledge. Participants also recorded their postcode, which was later recoded using Accessibility/Remoteness Index in Australia (ARIA) 2011 data, developed by the National Center for the Social Applications of Geographic Information Systems into the categories major cities, inner/outer regional, and remote/very remote [22].

### Ethics Approval

This study was approved by the University of Queensland Institutional Human Research Ethics Committee (approval number: 2015001652).

## Results

During the 30-day recruitment period, 498 participants were recruited to the Cool Runnings study through Facebook and Instagram advertisements.

### Participant Demographics

**Figure 2:**
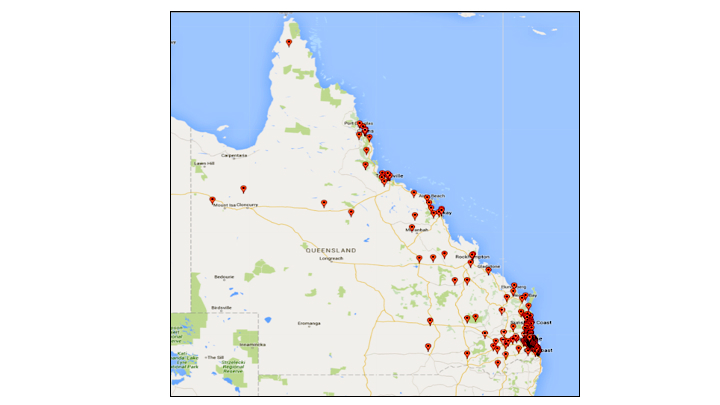
Map of Queensland (area 1,852,642 km^2^), Australia, showing the broad reach of study participants (marked by pins).

The demographic characteristics of recruited participants compared with mothers who birthed in Queensland in 2015 (the year the study was conducted), derived from the Queensland Perinatal Data Collection Report 2015 [23] is shown in Table 1. While statistical comparisons were not possible, these data indicated that participants recruited for this study were similar to the target population (mothers who gave birth in Queensland) on most characteristics (age group, marital status, country of birth, first-time mother), except smoking status. No comparable data were available for education level or ARIA. The location of usual residence was categorized using ARIA, developed by the National Center for the Social Applications of Geographic Information Systems. Each geographical area was allocated a score between 0 and 15, based on the (road) distance to nearby towns that provide services [24]. Scores were then allocated to the following categories: urban (major city: 0.0 to 0.2); peri-urban (inner regional: 0.2 to 2.4; outer regional: 2.4 to 5.92); and remote (remote: 5.92 to 10.53; very remote: 10.53 and greater). The broad reach of study participants is highlighted in Figure 2.

**Table 1:** Demographic characteristics of recruited participants (N=498) and Queensland population data for mothers in 2015.

Characteristic		Recruited participants, n (%)	Queensland mothers, n (%)
**Age**			
	18-24 years	89 (17.9%)	20%
	25-29 years	176 (35.4%)	28%
	30-34 years	161 (32.3%)	32%
	35-39 years	62 (12.4%)	16%
	40+ years	10 (2.0%)	4%
First-time mothers		216 (43.4%)	41%
**Marital status**			
	Married/defacto	416 (83. 5%)	84%
	Single	67 (13.5%)	14%
	Separated/divorced	13 (2.6%)	1.4%
	Current smoker	97 (19.5%)	12%
**Country of birth**			
	Australia	419 (84.1%)	74%
	New Zealand	23 (4.6%)	5%
	United Kingdom	23 (4.6%)	3%
	Other	33 (6.6%)	18%
**Highest education level**			N/A
	Less than Year 12	86 (17.3%)	
	Year 12 completion	131 (26.3%)	
	Advanced diploma/trade certificate	127 (25.5%)	
	University degree	112 (22.5%)	
	Post-graduate degree	42 (8.4%)	
**ARIA^a^**			N/A
	Urban (major cities)	238 (47.8%)	
	Peri-urban (inner/outer regional)	205 (41.2%)	
	Remote/very remote	49 (9.8%)	

^a^ARIA: Accessibility/Remoteness Index of Australia.

### Facebook Recruitment Outcomes

**Figure 3:**
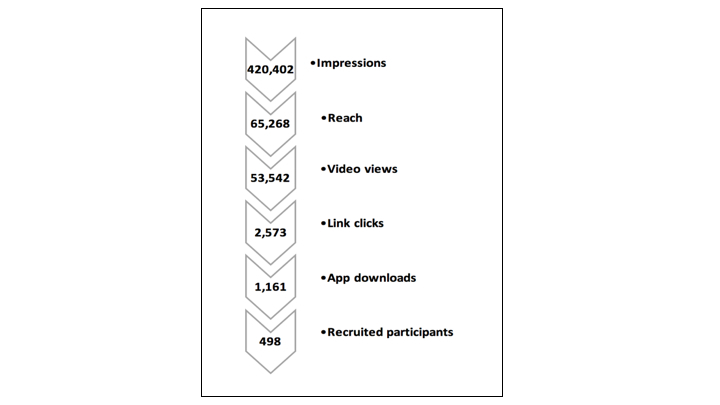
Flowchart of the recruitment process from Facebook impressions to recruited participants .

Facebook and Instagram advertisements generated 420,402 impressions and reached 65,268 people, generating 2573 link clicks and 1161 app downloads. There were 291 post reactions (like, love, etc.), 61 comments, and 164 shares. The cost of advertisements (ads) per 1000 impressions was Aus $16.39, per 1000 people reached Aus $105.40, and per recruited participant Aus $13.08. The recruitment process from ad impressions through to recruited participant is shown in Figure 3. Based on data from App Annie (San Francisco, USA), an industry standard app ranking and analytics company [25], in February 2016 the Cool Runnings app ranked number 48 in Australia for all educational app downloads.

**Figure 4:**
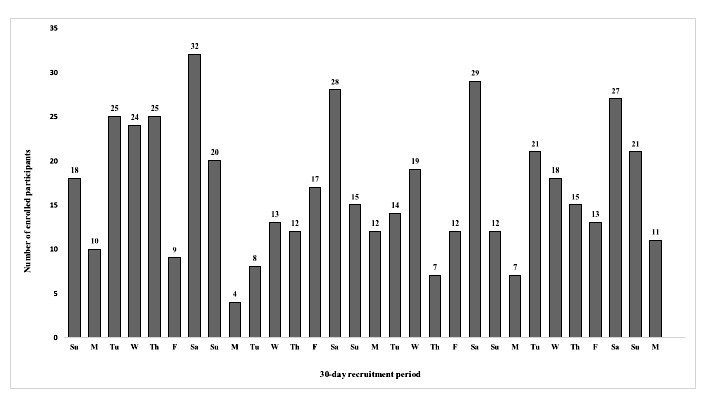
Number of participants enrolled each day of the 30-day recruitment period.

Of the 45 ad sets, 22 (49%, 22/45) were emotive-based (12 [55%, 12/22] videos, 6 [27%, 6/22] images, 4 [18%, 4/22] carousel), 16 (36%, 16/22) were incentive-based (6 [38%, 6/16] videos, 6 [38%, 6/16] images, 4 [25%, 4/16] carousel), and the remainder used mixed themes. Two emotive-based video ads were the most effective, resulting in 72.1% (359/498) of all participants recruited. Saturdays were the most effective day of the week for participant enrollment, and 5 to 11 PM was the most popular time of day with 55.0% (274/498) of enrollments occurring during these hours. The effect of advertisement optimization during each day of the recruitment period is shown in Figure 4. Once the advertisements started to be ineffective they were cut and the budget placed on the advertisements that were performing well.

Thirty-two adaptations of the ad copy were developed based on the variables listed earlier and split-tested. In the first 3 days of recruitment, 40 participants were recruited from 18 of the 32 adaptations. The advertisements that did not resonate with the targeted audience were removed and the budget reallocated to the advertisements that were performing well. This process was repeated until just 2 advertisements remained—both emotive-based videos on iOS and Android. The remaining budget was then allocated to these 2 advertisements. Individuals were removed from the advertising audience once they had been recruited. This saved advertising budget and stopped participants from continually seeing the recruitment messages. This ability to access Facebook’s analytics and real-time reports on the effectiveness of different images, message themes, and message wording allowed more effective and efficient use of time and resources.

## Discussion

Recruiting 498 eligible participants in 30-days for less than Aus $14 per participant demonstrated that recruiting using Facebook’s targeted paid advertising on its 2 platforms (Facebook and Instagram) is an efficient and cost effective method for recruiting mothers of young children to public health research programs.

### Targeted Advertisements

For this method of recruitment to be effective it is important to first understand how the targeted audience uses social media and what social media platforms are they using.

Facebook is the most widely used of the major social media platforms and its user base is most broadly representative of the population as a whole across age and gender [26,27]. However, it is important to note that some social media platforms are more popular among certain demographics. For example, in Australia Twitter is more popular among males and Pinterest is more popular among females; adults under 30 years prefer Instagram and Snapchat, while LinkedIn is more popular among older adults [28]. For targeting millennial mothers, Facebook and Instagram were an ideal choice given mothers active daily use on these platforms.

### Reach, Representativeness, and Cost

Accessibility to Facebook and Instagram’s large and diverse users address one of the challenges facing many research projects when it comes to recruiting—adequate size and representativeness of sample. The literature confirms targeted Facebook advertising has been effective in recruiting populations based on geographic location, age, and gender, but also specific, often hard-to-reach populations [29-31]. Mothers of young children were the focus of this recruitment strategy and are the most active users on Facebook [6,7]. The targeted advertisements for this study delivered participants from a variety of socio-economic, geographic, and educational backgrounds. There was good representation of mothers across the age groups and an almost equal split of premipara (first-time mothers) and multipara participants. These participants were largely representative of the target population (women who birthed in Queensland in 2015) with regard to age, marital status, being a first-time mother, and country of birth [23].

While the participants for this study were well represented on Facebook, Instagram, and many other social media platforms, it is important to note there are populations that are not so well represented on social media, such as older, economically disadvantaged, rural/remote, and less educated individuals [2]. However, research by the Pew Institute [32] shows these trends are changing. These issues and limitations also affect traditional recruitment samples.

A number of studies have compared social media recruitment with traditional recruitment methods in terms of cost and speed, with the majority showing social media to be more effective for both [2,33]. However, a review of 30 studies that compared social media with other recruitment methods reported only 12 (40%, 12/30) found social media to be the best recruitment method overall [3]. Social media recruitment is reported to be better for recruiting hard-to-reach populations. A systematic review by Thornton et al [2] reported the average cost per enrolled participant using Facebook recruitment was US $17 (range $1.36 to $110). Traditional recruitment methods can cost US $20 to $500 per participant, depending on the strategy and target population [34-36].

### Limitations

This recruitment strategy had some limitations. The social nature of Facebook increases the likelihood of snowballing, with individuals sharing the study advertisements with their Facebook friends, potentially leading to sampling bias. Another limitation is relying on information individuals provide on their Facebook and/or Instagram profile, which may not be correct or up-to-date. Some interested individuals who received the targeted advertisements were not eligible for recruitment as they no longer lived in Queensland but had not updated their profile information. Because some of the baseline questions for Cool Runnings were to determine knowledge about burn risks to children, we were unable to mention burn prevention to children specifically in the advertisements. This may have led to confusion in interested individuals. Finally, the initial advertisement sets did not specify that participants had to have at least 1 child aged between 5 to 12 months. This led to some interested individuals downloading the app and then finding they were ineligible once they read the participant information/consent page. This issue was rectified in the second week of recruitment.

To our knowledge, this is the first use of social media recruitment for an injury prevention campaign. Based on the reach, representativeness, cost, and speed of social media recruitment for this study, and as reported in the literature, this recruitment method would be beneficial for recruiting targeted populations at risk of specific injuries. It also has great potential for public health campaigns that want to reach and engage large numbers of people, whether it is to promote healthy behaviors, prevent disease, or reduce injuries.

### Conclusions

Recruiting via social media allowed a rapid and cost-effective recruitment of mothers of young children to an injury prevention campaign. The social, geographical, and economic diversity of the recruited participants demonstrates the power of social media recruitment as a positive option for studies needing to recruit hard-to-reach populations or representative study samples.

## Abbreviations

ARIA: Accessibility/Remoteness Index in Australia
RCT: randomized controlled trial
